# Reply to: holistic effort at curbing tobacco use essential in the US

**DOI:** 10.1016/j.lana.2023.100550

**Published:** 2023-07-11

**Authors:** Mohammad Ebrahimi Kalan, Noel T. Brewer

**Affiliations:** a School of Health Professions, Eastern Virginia Medical School, Norfolk, VA, United States; b Department of Health Behavior, Gillings School of Global Public Health, University of North Carolina, Chapel Hill, NC, United States; c Lineberger Comprehensive Cancer Center, University of North Carolina, Chapel Hill, NC, United States

Electronic nicotine delivery systems (ENDS) have transformed the tobacco products landscape in recent years. The market for ENDS has grown rapidly even as the number and types of these products have also grown.^1^ This growth in ENDS products coincided with the lack of consistent regulations and quality control, raising concerns about the safety and long-term health effects of ENDS, especially loopholes for youth access in ENDS delivery sales laws in the US.^2^^,^^3^ Our study of US adults^4^ showed high variability in using ENDS and cigarettes over time. We also found that an increase in ENDS use from 2015 to 2019 among adults led to a decrease [in an absolute term] in cigarette use.^4^ This calls for a comprehensive research and policy approach, as Saxena and Godfre highlighted.^5^ Future waves of the Population Assessment of Tobacco and Health (PATH) data will provide crucial information for regulatory bodies on the variability in ENDS and cigarette use, and whether the former can be a game changer for later. One concern is the potential for dual use, where individuals continue to use both ENDS and cigarettes. This pattern of use undermines the potential benefits of ENDS for quitting smoking, if any. In 2021,^6^ 4.5% of adults in the US reported using ENDS currently (i.e., used on some days or every day), with 29.4% of whom were also current cigarette smokers (i.e., dual use), 40.3% were former cigarette smokers, and 30.3% had never been cigarette smokers. Quitting smoking altogether, and eventually quitting ENDS use, remains the best option for improving health outcomes. A comprehensive approach is needed to benefit from ENDS and other cessation programs to curb combustible cigarette smoking, the number 1 cause of premature death worldwide. And then to wind down population-wide use of ENDS.

## Contributors

Mohammad Ebrahimi Kalan: Conceptualization, literature search, writing–original draft, writing-review & editing.

Noel Brewer: Conceptualization, literature search, writing–original draft, writing-review & editing.

## Declaration of interests

None.
